# Magnetic Induction Spectroscopy for Biomass Measurement: A Feasibility Study

**DOI:** 10.3390/s19122765

**Published:** 2019-06-20

**Authors:** Ziyi Zhang, Mohammed Ali Roula, Richard Dinsdale

**Affiliations:** 1 National Innovation Institute of Defense Technology, Academy of Military Science, Beijing 100071, China; 2 Faculty of Computing, Engineering and Science, University of South Wales, Pontypridd CF37 1DL, UK; richard.dinsdale@southwales.ac.uk

**Keywords:** magnetic induction spectroscopy (MIS), biomass, bio-impedance, conductivity, phase perturbation

## Abstract

Background: Biomass measurement and monitoring is a challenge in a number of biotechnology processes where fast, inexpensive, and non-contact measurement techniques would be of great benefit. Magnetic induction spectroscopy (MIS) is a novel non-destructive and contactless impedance measurement technique with many potential industrial and biomedical applications. The aim of this paper is to use computer modeling and experimental measurements to prove the suitability of the MIS system developed at the University of South Wales for controlled biomass measurements. Methods: The paper reports experimental measurements conducted on saline solutions and yeast suspensions at different concentrations to test the detection performance of the MIS system. The commercial electromagnetic simulation software CST was used to simulate the measurement outcomes with saline solutions and compare them with those of the actual measurements. We adopted two different ways for yeast suspension preparation to assess the system’s sensitivity and accuracy. Results: For saline solutions, the simulation results agree well with the measurement results, and the MIS system was able to distinguish saline solutions at different concentrations even in the small range of 0–1.6 g/L. For yeast suspensions, regardless of the preparation method, the MIS system can reliably distinguish yeast suspensions with lower concentrations 0–20 g/L. The conductivity spectrum of yeast suspensions present excellent separability between different concentrations and dielectric dispersion property at concentrations higher than 100 g/L. Conclusions: The South Wales MIS system can achieve controlled yeast measurements with high sensitivity and stability, and it shows promising potential applications, with further development, for cell biology research where contactless monitoring of cellular density is of relevance.

## 1. Introduction

**Figure 1:**
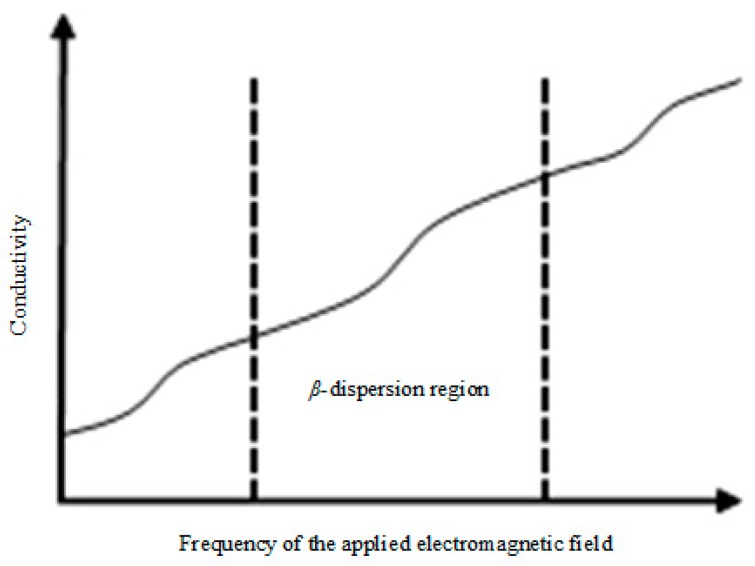
Sketch diagram of the conductivity spectrum for biological samples.

Bio-impedance versus the frequency of the applied electromagnetic field, known as the bio-impedance spectrum, is an important property of biological samples. It can be characterized by passive electrical properties of biological samples. For most non-magnetic biological samples, permittivity and conductivity are the two most important passive electrical properties, which are both frequency-dependent and can be used as a unique signature to distinguish different samples or different states within the same sample. The distinction of the bio-impedance spectrum mainly focuses on the magnitude or shape of the spectral line, especially in the *β*-dispersion region (the radiofrequency range from 1 kHz to 100 MHz) [1]. Figure 1 shows a sketch diagram of the conductivity spectrum for biological samples. As a matter of fact, in the *β*-dispersion region, the magnitude and shape of the sample’s impedance spectrum are associated closely with the sample’s cellular characteristics due to the polarization of cellular membranes, proteins, and other organic molecules, and this mechanism is the well-known Maxwell–Wagner effect [2,3,4]. Therefore, changes in the sample’s impedance spectrum can reflect changes in the ionic environment within the sample and changes in the sample’s inner structure at the cellular level. This principle makes the bio-impedance spectrum measurement a powerful tool in many applications [5,6,7]. 

Magnetic induction spectroscopy (MIS) is a new non-destructive and contactless technique that aims to measure the impedance spectrum (commonly known as the conductivity spectrum). Compared with the conventional electrode-based method, the MIS method can naturally eliminate errors caused by the electrode–sample interface and hence has drawn much attention in recent years. Scharfetter et al. [8] tried to measure the normalized conductivity spectrum of potato by using a system with a plan gradiometer as the detection coil. The same team coined the term ‘magnetic induction spectroscopy’ for the first time [9]. González et al. [10,11] attempted to use the technique in for the detection of detect brain and breast pathologies by measuring the phase shift spectrum and its relation to medically relevant characteristics in the medium. O'Toole et al. [12] further investigated the MIS system for in-line industrial usage. The conductivity spectra for agricultural produce (e.g., apples, potatoes, etc.) were measured and were found to compare well with the existing measurement data in the literature. The same team also studied its application in the classification of non-ferrous metals [13]. Li and Yan et al. [14,15] focused on intracerebral hemorrhage (ICH) detection using the magnetic induction phase shift (MIPS) method. Experiments on rabbits revealed that their MIPS system could detect ICH in real time and differentiate ischemic stroke from hemorrhagic stroke effectively. Their animal and human measurements showed that the MIS method has promising clinical prospects. Maimaitijiang et al. [16] went as far as experimenting with multichannel MIS system on animal phantom heads for the detection of brain hemorrhages. Zakaria et al. [17] proposed using the MIS method for fetal hypoxia using electromagnetic simulation results based on COMSOL Multiphysics confirmed the feasibility of this application. Wang et al. [18] constructed a ferrite-cored MIS gradiometer to measure the impedance spectrum of cervical tissues. Lyons et al. [19] focused on the hardware and software design to achieve the high precision of phase detection in the MIS system. Ma et al. [20] studied the feasibility of imaging the frequency-dependent permeability of ferromagnetic materials combining the MIS and tomographic method. 

The precise and real-time in situ measurement of biomass is important in many biotechnology processes, such as cultivation or food processes [21,22,23]. It requires a measurement system with a robust feature to resist artificial and environmental noises, especially in cases of low sample cell concentrations. Among numerous methods, dielectric spectroscopy (DS) based on probes or electrodes has been a common choice for biomass measurement and monitoring due to its advantages of fast, simple, and low-cost features [24,25,26]. However, invasive or contact sensors in these DS systems could lead to serious measurement errors and even contaminate and damage samples under test. Aiming to address these difficulties, in this paper, we focus on the biomass measurement used in the food industry application using the non-destructive and non-contact MIS technique. We evaluate the feasibility of biomass measurement using the South Wales MIS system [27]. The sensitivity and accuracy of the system are investigated by conducting measurements with saline solutions and yeast suspensions with lower concentrations 0–1.6 g/L and 0–20 g/L, respectively. To verify the validity of the measurements, we also simulate the measurements with saline solutions in the commercial electromagnetic simulation software CST and prepare two different types of yeast suspensions for investigation.

## 2. Methods

### 2.1. Description of the MIS Measurement Principle

**Figure 2:**
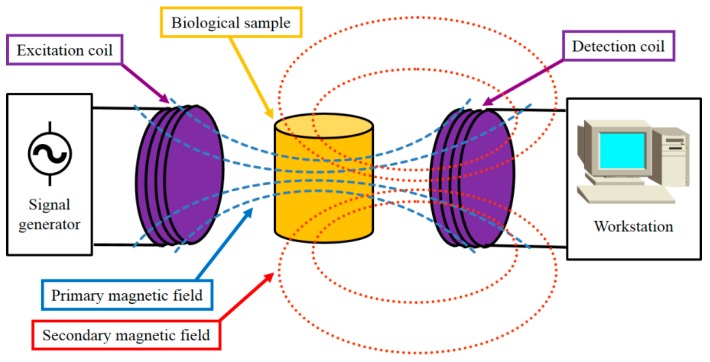
Measurement Principle of the magnetic induction spectroscopy (MIS) Method.

The measurement principle of the MIS method is illustrated in Figure 2. A pair of coils, namely an excitation coil and a detection coil, make up the MIS coil system. The excitation coil is connected with the signal generator, and the detection coil is connected with the workstation, which can process measured signals, compute data, and output results. When a sinusoidal alternating current from the signal generator is applied to the excitation coil, the excitation coil radiates an alternating magnetic field *B*_0_ (primary magnetic field) into the space, and hence, a primary voltage *V*_0_ can be measured in the detection coil. Then, a biological sample under test is placed between the excitation coil and the detection coil. The sample is assumed to be a homogeneous, linear, isotropic, and non-magnetic medium. Under the primary magnetic field, the eddy current is induced within the sample and a secondary magnetic field is generated. The magnetic field perturbation Δ*B* can produce a voltage perturbation Δ*V* in the detection coil. It can be derived that

(1) Δφ=Im(ΔBB0)=Im(ΔVV0)∝fσ,

where Δ*φ* denotes the phase perturbation in the detection coil due to the presence of the sample, Im refers to the imaginary part, *f* is the frequency of the excitation current, and *σ* is the conductivity of the sample. Equation (1) shows that the phase perturbation is proportional to the product of the frequency and the conductivity, meaning that the phase perturbation contains information on the dielectric dispersion property (conductivity) of the sample. The proportional coefficient would depend on the geometry of the MIS coil system and the sample’s geometry and position within the system.

For multi-frequency MIS measurements, as the conductivities of the biological samples are all frequency-dependent, Equation (1) can be expressed as follows:

(2) $$\sigma \left(f_{i}\right)=K\frac{\Delta \varphi_{i}}{f_{i}},$$

where *f_i_* (*i* = 1, 2, …) is the *i*th frequency component of the excitation currents, Δ*φ_i_* is the measured phase perturbation at *f_i_*, *σ* (*f_i_*) represents the conductivity of the sample at *f_i_*, and *K* denotes the proportional coefficient, which is constant. The conductivity data calculated from Equation (2) form a curve called the conductivity spectrum, which shows the relationship between the conductivity of the sample and the excitation frequency.

### 2.2. The South Wales MIS System

**Figure 3:**
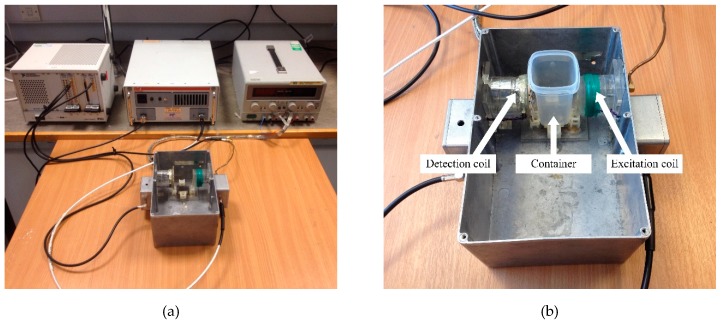
The South Wales MIS system. (**a**) Photograph of the measurement system, and (**b**) photograph of the coil system.

The South Wales MIS system used in this paper is shown in Figure 3. The coil system was fixed inside an aluminum enclosure, which decreased the interference from electromagnetic fields in the environment. The excitation coil and the detection coil were solenoids and placed coaxially. The numbers of turns for the excitation coil and the detection coil were 8 and 2, respectively. Both the coils had a radius of 22.5 mm, and their distance was 65 mm. A plastic container was designed to hold the biological sample. The geometry of the container was approximatively cuboid with dimensions of 37 mm × 37 mm × 65 mm. The container could be fixed on a small base at the bottom of the enclosure. The distances from the container to the excitation coil and the detection coil were 18 mm and 10 mm, respectively. Electric field shielding structures were added into the coil system to eliminate the electric field coupling between the excitation coil and the detection coil. A NI PXI-5412 arbitrary signal generator generated eight sinusoidal currents at 0.205, 0.405, 0.805, 1.605, 3.205, 6.405, 12.8, and 20.1 MHz frequencies. The current signals were then amplified by an AR KAA1020 power amplifier with an output power of 25 Watts that was applied to the excitation coil. A NI PXI-5105 digitizer was used to digitize measured signals, and the corresponding phase perturbations could be calculated by the Fast Fourier Transform (FFT) algorithm in the LabVIEW software.

When the sample under test was placed in the container, the phase perturbations could be measured by the system. However, in Equation (2), *K* needed to be estimated before calculating the conductivity data. Theoretically, for samples such as solutions or suspensions, which fill the container fully and homogeneously, they should have the same *K*, because all the factors affecting *K* have not changed, and in this case, *K* could be calculated by using a sample with known conductivity (e.g., saline solution) at a specific frequency chosen from *f_i_*. For samples that have irregular shapes and cannot fill the container suitably, their absolute conductivity data cannot be obtained directly due to the difficulty in calculating *K.* However, it is still possible to get their relative conductivity data *σ_r_* (*f_i_*) by using Equation (3)

(3) $$\sigma_{r}\left(f_{i}\right)=\frac{\Delta \varphi_{i}}{f_{i}}/\frac{\Delta \varphi_{0}}{f_{0}},$$

where *f*_0_ is a specific frequency chosen from *f_i_* and Δ*φ*_0_ is the phase perturbation at *f*_0_.

### 2.3. MIS Measurements and Simulations with Saline Solutions

A saline solution is the preferred sample to test the detection performance of an MIS system due to its constant conductivity with respect to the frequency. We added table salt to the deionized water to produce saline solutions with different concentrations. The temperature was kept at room temperature (about 20 °C) during the measurements. The experiments were composed of two steps: (i) measuring the saline solutions with concentrations of 0, 2, 4, 6, and 8 g/L to obtain a general detection performance of the system, and (ii) conducting measurements on saline solutions with lower concentrations at a range of 0–1.6 g/L to study the sensitivity of the system. The concentrations and the corresponding conductivities of the saline solutions used in the measurements are shown in Table 1. In order to obtain the conductivity spectra of the saline solutions, *K* was calculated based on the measurements of 8 g/L saline solution at 0.805 MHz.

**Figure 4:**
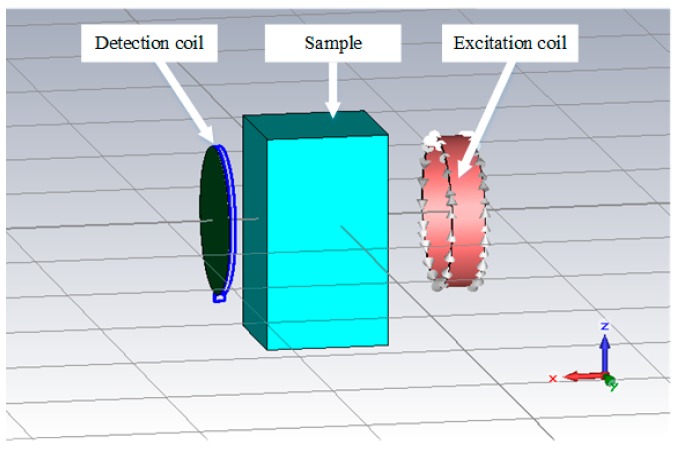
Models in CST Simulations.

In order to validate the reliability of the measurement results, we also conducted calculations to simulate measurements with saline solutions in the commercial electromagnetic simulation software CST (version 2016, CST AG). As shown in Figure 4, the excitation and detection coils were constructed, and a cuboid-shaped model was used as the container with the saline solution. All the parameters used in the simulations were based on the actual values in the measurements. The simulations were composed of two steps. The electromagnetic studio was selected, and the frequency domain full wave solver was used. CST applies the finite element method to solve time-harmonic Maxwell’s equations to obtain the electromagnetic field in the region of interest. After the electromagnetic field was determined, the phase perturbations at the detection coil were computed, and the conductivity spectra of the saline solutions were obtained by using Equation (2). *K* was also calculated based on the measurements with 8 g/L saline solution at 0.805 MHz.

### 2.4. Measurements with Yeast Suspensions

Yeast is a good medium for experimentation on other cellular suspensions, as it has a relatively homogeneous conductivity. Two methods were used for yeast suspension preparation. The first involved adding the dried yeast to the saline solution with a conductivity of 0.6 S/m. In this method, yeast suspensions with concentrations of 0, 1, 2, 5, 10, 20, 50, 100, and 200 g/L were cultured. In the second method, yeast growth media were used to develop yeast suspensions with concentrations of 0, 1, 5, 10, and 20 g/L. We aimed to check the system’s ability to distinguish yeast suspensions with lower concentrations in this way. The details of the growth media for the yeast are given in Table 2, in which the yeast extract typically contains all the amino acids necessary for growth, and peptone is the most widely used source of nitrogen. The measurements were conducted at room temperature (about 20 °C). As discussed in the measurement principle, the constant *K* for the yeast suspensions should be equal to that for the saline solutions.

## 3. Results

### 3.1. Measurements and Simulations with Saline Solutions

**Figure 5:**
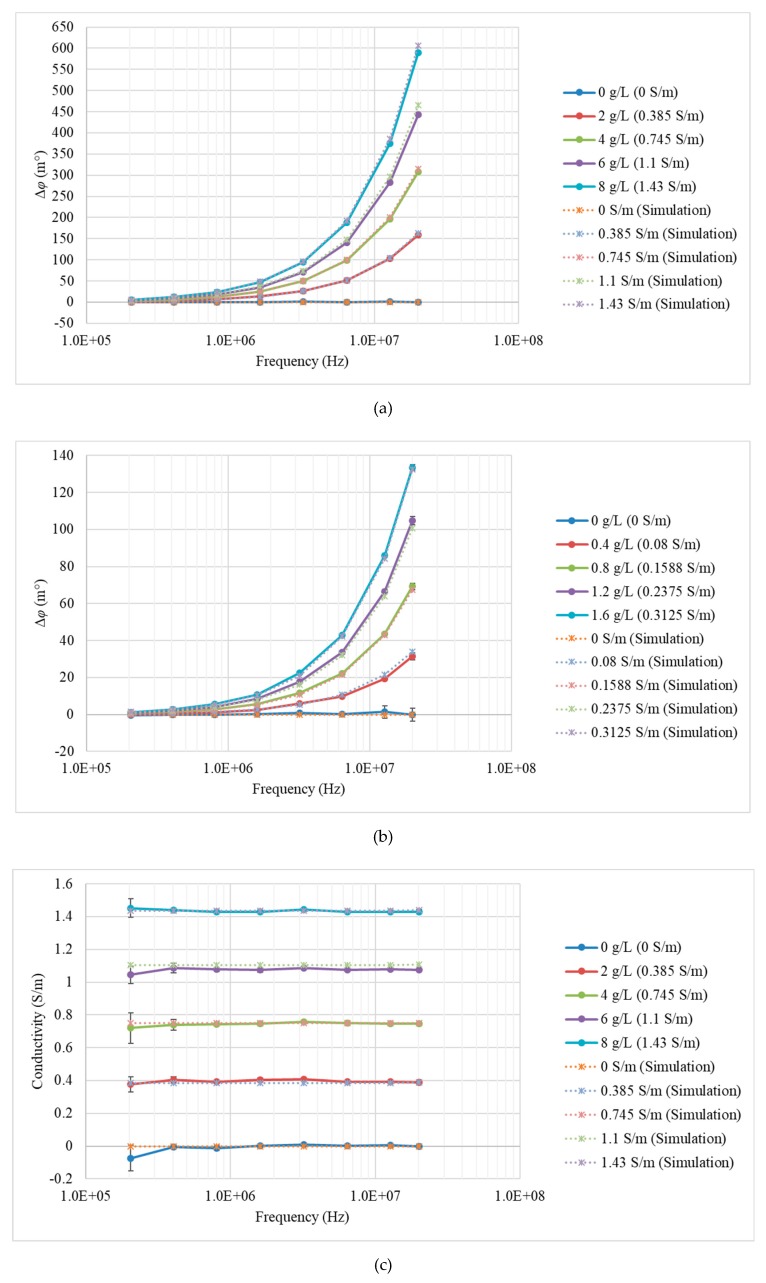

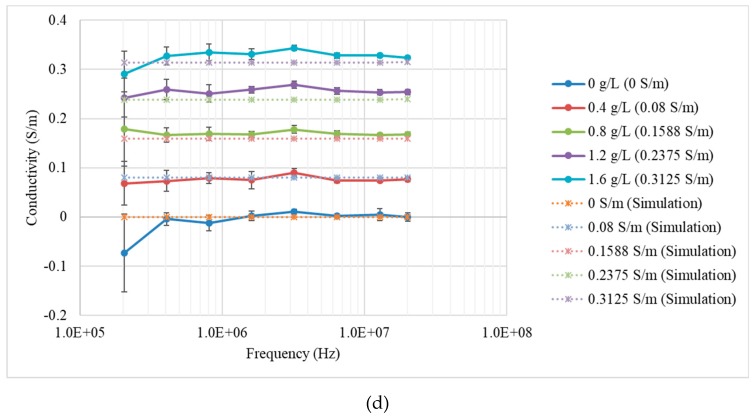
Measurement (solid curves) and simulation (dash curved) results for saline solutions. (**a**) Phase perturbation spectrum with concentrations of 0, 2, 4, 6, and 8 g/L; (**b**) phase perturbation spectrum with concentrations of 0, 0.4, 0.8, 1.2, and 1.6 g/L; (**c**) conductivity spectrum with concentrations of 0, 2, 4, 6, and 8 g/L; and (**d**) conductivity spectrum with concentrations of 0, 0.4, 0.8, 1.2, and 1.6 g/L. The error bars for the measured data were calculated based on ± 1 SD.

**Figure 6:**
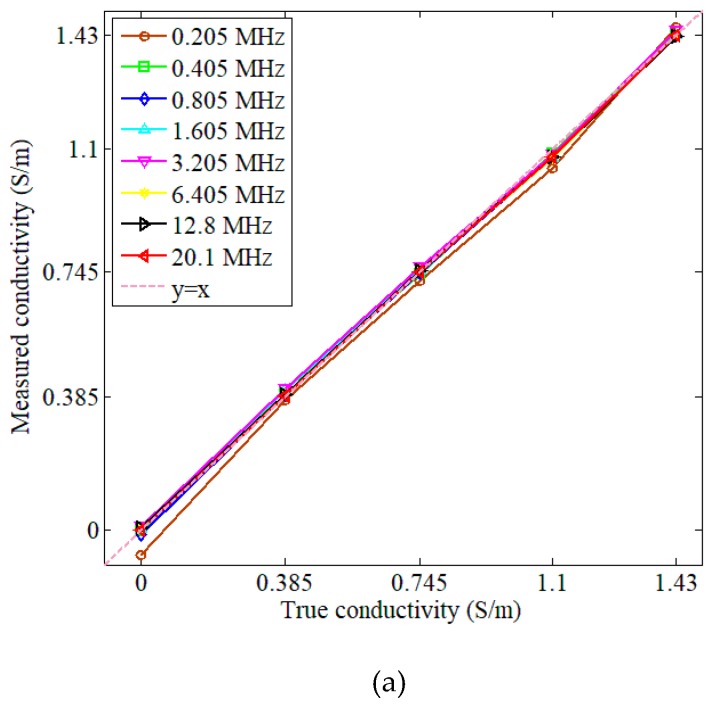

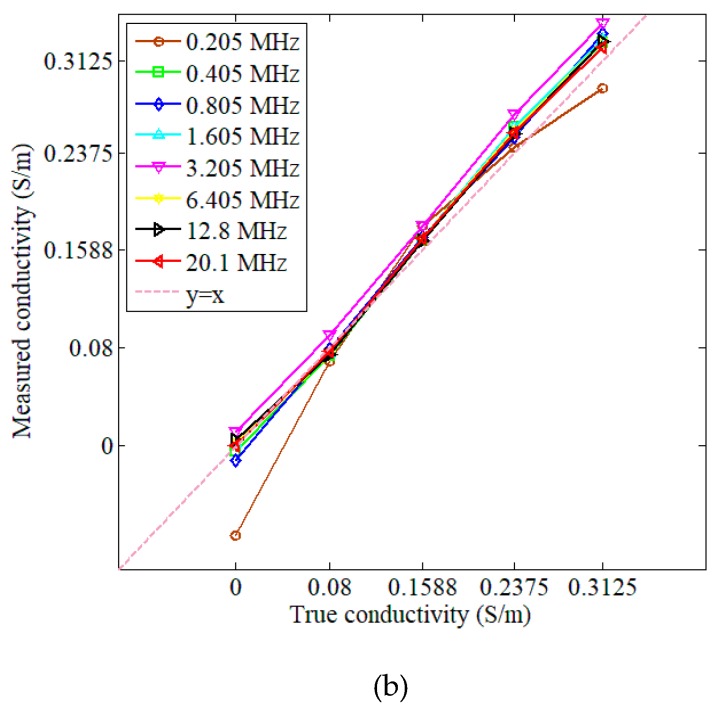
Correlation curves at eight frequency components for (**a**) saline solutions prepared in step (i) with concentrations of 0, 2, 4, 6, and 8 g/L and (**b**) saline solutions prepared in step (ii) with concentrations of 0, 0.4, 0.8, 1.2, and 1.6 g/L. On the correlation curves, the *x*-axis is the true conductivity, and the *y*-axis is the measured conductivity.

Each measurement was conducted 10 times and then averaged to produce recorded values. Phase perturbations were calculated for all eight excitation frequencies. The standard deviation (SD) of each measured data was also calculated. The value of *K* was calculated as 48,817 for measurements, and for simulations this value was 47,513. Figure 5 shows the measured (solid curves) and simulated (dash curves) phase perturbation and conductivity spectra of the saline solutions with concentrations in the range of 0–8 g/L. The results for step (i) are shown in Figure 5a,c, and the results for step (ii) are shown in Figure 5b,d. The correlation curves for the MIS system were plotted using saline solutions with known conductivities to evaluate the mismatch between the true conductivity and the measured conductivity, as shown in Figure 6. Values of the linear correlation coefficient and the norm of residuals for the data in each correlation curve were calculated and listed in Table 3.

### 3.2. Measurements with Yeast Suspensions

**Figure 7:**
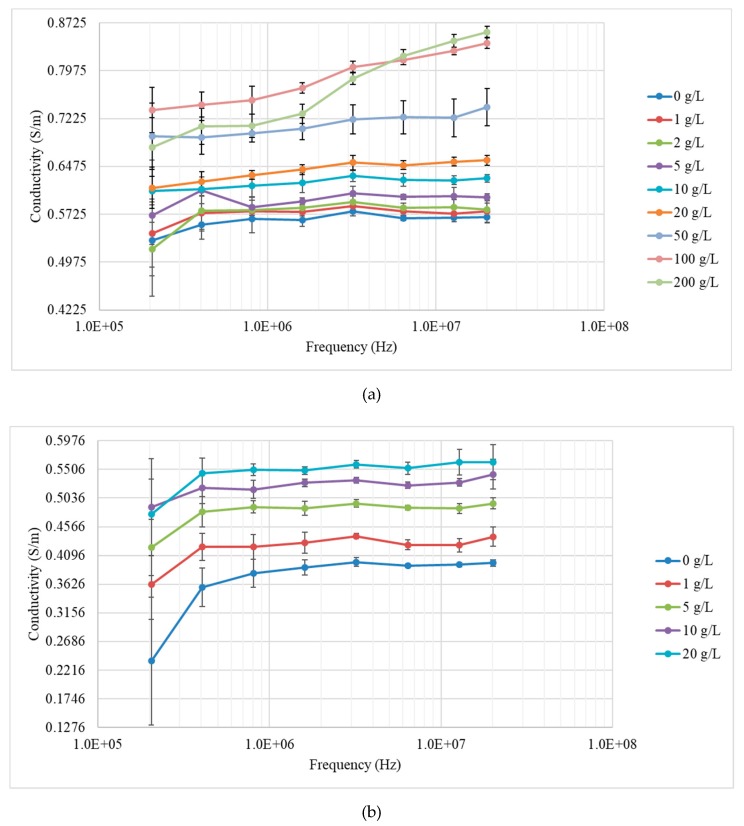
Measurement results for the yeast suspensions developed via the first (**a**) and second (**b**) preparation method. The error bars represent ± 1 SD.

A similar protocol was used as for saline solution measurements. Each measurement for the yeast suspensions was repeated 10 times. The yeast suspension was slowly and evenly stirred in order to make it homogeneous before MIS measurement. The value of *K* for the yeast suspensions was 48,817, which was the same as that of the saline solutions. The averages which could effectively reduce system and human errors used in Equation (2) to obtain the conductivity data. The conductivity spectra with error bars equal to ± 1 SD for the yeast suspensions for the two preparation methods are shown in Figure 7a,b, respectively.

**Figure 8:**
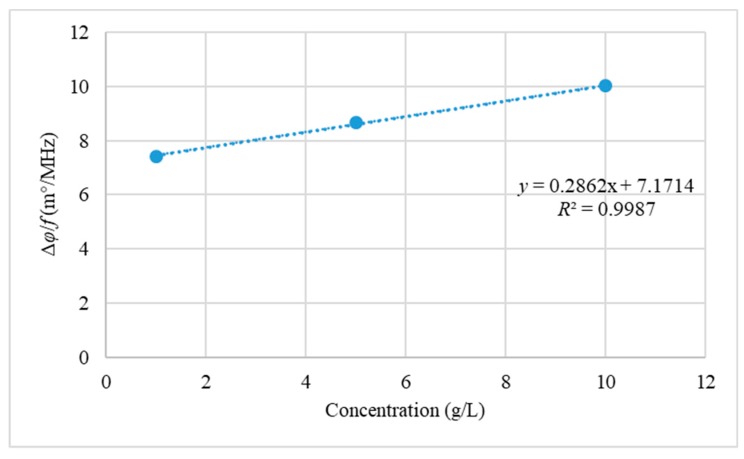
Calibration curve for the yeast suspensions prepared via the second method at 0.205 MHz.

We estimated the limit of detection (LoD) and the limit of quantitation (LoQ) for the yeast suspensions prepared via the second method at the lowest frequency component (0.205 MHz), as this type of yeast suspension contained living cells, which is of general interest in biomass. The LoD is the lowest analyte concentration likely to be detected to produce a response that can be reliably distinguished from that of a blank. The LoD is approved by estimations based on the standard deviation of the response *δ* and the slope *S* of the calibration curve at the levels approaching the limits according to the equation LoD = 3.3 × (*δ*/*S*). The LoQ is the lowest analyte concentration that can be measured with predefined and acceptable inaccuracy. The LoQ can be estimated according to the equation LoQ = 10 × (*δ*/*S*) [28]. Figure 8 shows the calibration curve for the yeast suspensions prepared in the second method at 0.205 MHz.

## 4. Discussion and Conclusions

For saline solutions, the relationship between the phase perturbation and the frequency could be linear, as the conductivities of saline solutions are independent of the frequencies. This linear characteristic is explained visually in Equation (1), and it is not in contradiction with the exponential characteristic (see Figure 5a,b), as the frequency axis in Figure 5 is plotted in a logarithmic scale. On the whole, the MIS system exhibited good performance, distinguishing the saline solutions at different concentrations even in the small range of 0–1.6 g/L. The conductivity spectra of the saline solutions were all flat curves with amplitudes almost equal to the true values. The CST simulation results agreed well with the measurement results, verifying the validity of actual measurements. 

The correlation curves in Figure 6 demonstrated good agreement between the true conductivity and the measured conductivity. All the correlation curves fluctuated closely around the standard line *y* = *x*. In Table 3, the values of the linear correlation coefficients were all close to unity. For step (i), the values of the norm of the residuals varied in the range of 0.0192–0.0601, with the maximum at 0.205 MHz. For step (ii), the values of the norm of the residuals varied in the range of 0.0046–0.0597, with a maximum at 0.205 MHz. In fact, the mismatch may be worse at the lower frequency components, and larger for solutions or suspensions with lower concentrations than higher concentrations.

In Figure 7, regardless of the preparation method used, the amplitude of conductivity showed an increasing trend, as the concentration of the yeast suspension increased. The conductivity spectrum of the yeast suspensions presented excellent separability between different concentrations, confirming that the MIS system can distinguish yeast suspensions with lower concentrations well. As shown in Figure 7a, the conductivity curves showed a dielectric dispersion property (S shape), which was heightened at concentrations higher than 100 g/L.

The calibration curve in Figure 8 shows Δ*φ*/*f* versus the concentration for the yeast suspensions prepared in the second method, and the curve was found to be linear within the concentration range of 1–10g/L. The LoD and LoQ were estimated at the lowest frequency component, because the MIS system may have experienced more noise in this condition. The estimated values of the LoD and LoQ were 0.76 g/L and 2.32 g/L, respectively. It should be noted that the calculations of the LoD and LoQ were approximate and rough. For more precise outcomes, more samples of yeast suspensions with different concentrations in the range of 1–10 g/L should be prepared and measured.

It should also be noted that the variance in repeated measurements (see the SD) was larger at the lower frequency components in all the measurements with the saline solutions and yeast suspensions. It can be seen from Equation (1) that the measurement precision for the phase perturbation was associated with the lower frequency components of the MIS system. However, the measured phase perturbations at lower frequencies had relatively low amplitudes, and these weak signals were easily interfered with by the noise from the system or humans, resulting in a low signal-to-noise ratio (SNR). In order to improve the SNR, on the one hand, more repeated measurements should be conducted to reduce random errors more effectively. On the other hand, the container with the samples under test should be placed in the MIS coil system slightly and carefully to avoid noise as much as possible during the measurements.

A limitation to scaling up to industrial applications is that the typical tank reactors are made of stainless steel, which would significantly interfere with excitation and detection signals. Another limitation is the sensitivity of the measurements to movements, which would prevent the steering and mixing expected in a realistic bioreactor. In conclusion, the South Wales MIS system is capable of taking sensitive and robust measurements of saline solutions and yeast suspensions with lower concentrations, demonstrating good detection performance with respect to biomass measurement, which suggests that, with further development, this system could potentially be used in contactless monitoring of cell density in several applications, such as in biomass, food processing, and biology cell research.

## Figures and Tables

**Table 1:** Concentrations and corresponding conductivities of the saline solutions used in the measurements.

Concentration (g/L)	Conductivity (S/m)
0	0
0.4	0.08
0.8	0.1588
1.2	0.2375
1.6	0.3125
2	0.385
4	0.745
6	1.1
8	1.43

**Table 2:** Growth media for yeast.

Composition	Concentration (g/L)
Yeast extract	10
Peptone	20
Glucose	20

**Table 3:** Values of the linear correlation coefficient and the norm of residuals for the data in each correlation curve.

Frequency (MHz)	Step (i)		Step (ii)	
	Linear Correlation Coefficient	Norm of Residuals	Linear Correlation Coefficient	Norm of Residuals
0.205	0.9987	0.0601	0.9788	0.0597
0.405	0.9997	0.0254	0.9990	0.0119
0.805	0.9998	0.0223	0.9998	0.0046
1.605	0.9997	0.0282	0.9990	0.0118
3.205	0.9997	0.0266	0.9996	0.0073
6.405	0.9997	0.0245	0.9990	0.0119
12.8	0.9998	0.0192	0.9989	0.0118
20.1	0.9998	0.0204	0.9993	0.0095
